# Breast cancer in patients with germline *TP53* pathogenic variants have typical tumour characteristics: the Cohort study of *TP53* carrier early onset breast cancer (COPE study)

**DOI:** 10.1002/cjp2.133

**Published:** 2019-05-23

**Authors:** Kate Packwood, Guy Martland, Matthew Sommerlad, Emily Shaw, Karwan Moutasim, Gareth Thomas, Adrian C Bateman, Louise Jones, Linda Haywood, D Gareth Evans, Jillian M Birch, Ohud A Alsalmi, Alex Henderson, Nicola Poplawski, Diana M Eccles

**Affiliations:** ^1^ Faculty of Medicine University of Southampton Southampton UK; ^2^ Cellular Pathology Department Poole Hospital NHS Foundation Trust Poole UK; ^3^ Cellular Pathology Department University Hospital NHS Foundation Trust Southampton UK; ^4^ Centre for Tumour Biology Department, Barts Cancer Institute Queen Mary University of London London UK; ^5^ Department of Genomic Medicine, Division of Evolution and Genomic Science University of Manchester Manchester UK; ^6^ School of Biological Sciences, Faculty of Biology, Medicine and Health University of Manchester Manchester UK; ^7^ Northern Genetics Service Newcastle upon Tyne Hospitals Newcastle UK; ^8^ Discipline of Paediatrics, Adelaide Medical School University of Adelaide Adelaide Australia

**Keywords:** breast cancer, TP53 pathogenic variant, germline, stroma

## Abstract

Germline *TP53* pathogenic variants are rare but associated with a high risk of cancer; they are often identified in the context of clinically diagnosed Li–Fraumeni syndrome predisposing to a range of young onset cancers including sarcomas and breast cancer. The study aim was to conduct a detailed morphological review and immuno‐phenotyping of breast cancer arising in carriers of a germline *TP53* pathogenic variant. We compared breast cancers from five defined groups: (1) *TP53* carriers with breast cancer (*n* = 59), (2) early onset HER2‐amplified breast cancer, no germline pathogenic variant in *BRCA1/2* or *TP53* (*n* = 55), (3) *BRCA1* pathogenic variant carriers (*n* = 60); (4) *BRCA2* pathogenic variant carriers (*n* = 61) and (5) young onset breast cancer with no known germline pathogenic variant (*n* = 98). Pathologists assessed a pre‐agreed set of morphological characteristics using light microscopy. Immunohistochemistry (IHC) for HER2, ER, PR, p53, integrin alpha v beta 6 (αvβ6) integrin, α‐smooth muscle actin (α‐SMA) and pSMAD2/3 was performed on tissue microarrays of invasive carcinoma. We confirmed a previously reported high prevalence of HER2‐amplified, ductal no special type invasive breast carcinoma amongst known *TP53* germline pathogenic variant carriers 20 of 36 (56%). Furthermore we observed a high frequency of densely sclerotic tumour stroma in cancers from *TP53* carriers (29/36, 80.6%) when compared with non‐carriers, 50.9% (28/55), 34.7% (50/144), 41.4% (65/157), 43.8% (95/217) in groups 2–5 respectively. The majority of germline *TP53* gene carrier breast tumours had a high intensity of integrin αvβ6, α‐SMA and pSMAD2/3 expression in the majority of cancer cells. In conclusion, aggressive HER2 positive breast cancers with densely sclerotic stroma are common in germline *TP53* carriers. High levels of αvβ6 integrin, α‐SMA and pSMAD2/3 expression suggest that the dense stromal phenotype may be driven by upregulated transforming growth factor beta signalling.

## Introduction

The importance of functional wild‐type p53 protein is exemplified by the fact that somatic pathogenic variants in *TP53* are detected in more than 50% of all cancer types, particularly more aggressive sub‐types, which constitute 28% of breast cancers 1, 2. Germline *TP53* pathogenic variants are rare and predispose to early onset breast cancer which, in combination with other tumour types such as malignant brain tumours and sarcoma, is recognised clinically as a manifestation of Li–Fraumeni syndrome (LFS) 3, 4. *TP53* encodes the p53 protein which has been referred to as ‘the guardian of the genome’ due to its crucial function in maintaining cellular homeostasis including cell cycle arrest, apoptosis, angiogenesis, metabolism, DNA damage and senescence in response to a number of genotoxic stressors 5.

It has been recognised for over two decades that breast cancers associated with *BRCA1* pathogenic variants are predominantly triple receptor (ER, PR and HER2) negative, high grade invasive ductal carcinomas with high numbers of tumour infiltrating lymphocytes (TILs) 6, 7, 8, 9, 10. More recently we reported a preliminary observation that germline *TP53* pathogenic variant carriers more commonly developed HER2 positive invasive breast cancer when compared to sporadic cases 9. Our preliminary observation has been confirmed by others 11, 12, 13. In this study, we describe an extended morphological and immunohistochemical characterisation of a larger cohort of early onset breast cancer patients with a germline *TP53* pathogenic variant. We were interested to describe not only the tumour features but also the characteristics of the tumour microenvironment (TME), particularly the stroma and presence or absence of TILs given the growing interest in the therapeutic barriers and opportunities of the TME.

## Methods

### Patients and samples

Formalin fixed paraffin embedded (FFPE) breast cancer tissue blocks were used for this study.

Group 1: The COPE study (Cohort study of *TP53* pathogenic variant carriers in early onset breast cancer) acquired breast cancer tissue blocks from patients with a known germline *TP53* pathogenic variant and breast cancer (multicentre research ethics committee approval (09/H0501/85) (Table 1).

**Table 1:** Cohorts and recruitment eligibility: patient selection and eligibility for groups 1–5: *TP53*, *BRCA1* and *BRCA2* gene carriers, HER2+and YBC with no underlying germline pathogenic variant

Cohort	COPE	POSH			
Total no. of patients	45	2956			
Group	1	2	3	4	5
Selection criterion	Germline *TP53*	No germline pathogenic variant HER2+	Germline *BRCA1*	Germline *BRCA2*	No germline pathogenic variant YBC
No. of patients	45	55	60	61	98
No. of reads	45	55	138	157	217
Morphological review as part of the COPE study*	✓	✓			
Morphological review as part of the POSH study**			✓	✓	✓
TMAs	✓	✓			

* COPE morphology review (three breast histopathologists).

** POSH morphology review involving 13 breast histopathologists described in Shaw *et al* 22.

Group 2: Tissue blocks were selected from young breast cancer (YBC) cases participating in the Prospective study of Outcomes in Sporadic versus Hereditary breast cancer (POSH) for comparison 14. POSH cases were selected based on the following criteria – tissue block available, no known pathogenic variant in *BRCA1*, *BRCA2* or *TP53*, invasive breast cancer reported as HER2 positive with associated ductal carcinoma *in situ* (DCIS) (group 2, *n* = 55). Ethical approval for POSH was granted in 2000 by South West MREC (00/6/69).

H&E‐stained sections for cases in groups 1 and 2 were assessed independently by two pathologists (GM, MS) for a range of morphological features (Table 1); discrepancies were resolved by a third pathologist (AB).

Groups 3–5: Data were available from a previously published morphological review comparing digital and conventional (glass slide) microscopy reading amongst cases from the POSH study 15. This additional dataset was therefore included for comparison of *TP53* germline pathogenic variant carriers with other high risk breast cancer susceptibility gene carriers and with young onset non‐carriers. Group 3 comprised known *BRCA1* carriers, group 4 known *BRCA2* carriers and group 5 YBC with no underlying germline pathogenic variant.

For groups 3–5, cases were randomly distributed between 13 histopathologists who assessed pre‐specified morphological features using conventional (glass slide) microscopy or a digital interface 15. The submitted reports from conventional microscopy were extracted for each reported case. The POSH cases were reported by more than one pathologist (1–3 per case) so, for the purposes of statistical analyses, we treated each report as a separate case. There were 138 pathologists' reports for *BRCA1* carriers, 157 for *BRCA2* and 217 for early onset non‐carriers.

### Morphological assessment

Morphological assessment was performed on 4 μm thick sections cut using a microtome (Leica Biosystems, Milton Keynes, Bucks, UK) from FFPE breast tumours. Sections were mounted on Superfrost Plus™ Adhesion Microscope Slides (ThermoFisher Scientific, Waltham, MA, USA). Slides were stained using H&E on an automated CoverStainer (Dako UK Ltd, Ely, Cambridge, UK).

### Tissue microarray construction

H&E sections were reviewed and 1 mm cores of three representative invasive and two representative *in situ* areas were taken from the corresponding tissue blocks. Cores were inserted into a new recipient paraffin block using a tissue arrayer (Alphelys Minicore^(R)^3, Langevin, France). 4 μm sections were cut from each TMA using a microtome (Leica).

### Immunohistochemistry

To further characterise breast cancer cases with germline *TP53* pathogenic variants (group 1), HER2, ER, PR and p53 IHC was performed on TMA sections using an automated system. For HER2, ER and PR IHC, the Roche Diagnostics Limited, West Sussex, UK antibody with the Ventana Benchmark XT platform and the Ultraview‐Universal DAB detection Kit were used. For p53 the Dako antibody was used with Dako PT link for antigen retrieval, a Dako Autostainer Link 48 staining platform and the Envision FLEX detection system. IHC for integrin alpha v beta 6 (αvβ6), α‐smooth muscle actin (α‐SMA) and pSmad2/3 was performed on TMA sections using a method described previously 16. For COPE cases with HER2 positive results, *in situ* analysis was conducted on whole tumour sections: dual hapten, dual chromagen *in situ* hybridisation (DDISH) for *HER2*/Chromosome 17 using the Roche Ventana INFORM kit performed on the Ventana Ultra platform as per manufacturer recommendations. All antibodies were optimised using national diagnostic standards (UK NEQAS central office, Sheffield, S Yorks, UK).

A further available dataset comprising ER, PR, HER2 and p53 scores from 25 TMAs (1260 cases) from the POSH cohort provided the typical frequency of these markers in YBCs.

### Scoring and statistical analysis

HER2 was scored as between 0 and 3 according guidelines used in routine clinical practice based on staining intensity, completeness of membrane expression and the proportion of cells stained 17; a score of either 2+ on IHC and a subsequent positive DDISH test or 3+ on IHC alone are considered positive. ER and PR were evaluated using the Allred scoring system where positive was defined as a score of ≥3 18. p53 was scored using a semi‐quantitative modified McCarthy ‘H’ score; the modified scoring system gave a maximum score of 7 based on the proportion of cancer cells staining positive (1 = <25%, 2 = 25–50%, 3 = 50–75%, 4 = >75%) and the strength of staining intensity (1 = weak, 2 = moderate, 3 = strong) 19, 20. αvβ6 and α‐SMA were scored based on the strength of staining intensity (1 = weak, 2 = moderate, 3 = strong) as described by Marsh and colleagues for αvβ6 scoring 16. pSMAD2/3 was scored using the same method as p53. Scoring was manual (two pathologists reaching a consensus via simultaneous viewing of each core).

For pSMAD2/3, the digital pathology Halo Image Analysis software was trained and used. Each core was checked for tissue content, a classifier (evaluation of tissue and background in the image) and a mark‐up (showing individual cell by cell scoring). Cores were excluded where they were incomplete, contained normal tissue or where the software was unable to score the material.

Summary statistics were used to describe the morphological characteristics of the cases. IBM SPSS Statistics program (IBM United Kingdom Limited, Portsmouth, Hampshire, UK) was used for Pearson Chi‐square, Fisher's exact and Wilcoxon signed rank tests. Pearson Chi‐square was used when the number of unpaired cases in each group was >5, Fisher's exact test was used when the number of unpaired cases in each group was <5 and the Wilcoxon signed rank test when testing statistical significance in paired data sets with a skewed distribution of data.

## Results

The COPE study recruited samples from 59 patients with a confirmed germline *TP53* pathogenic variant. Fourteen cases had insufficient tumour for the study and were excluded. Of the remaining 45 cases, 36 contained invasive carcinoma and the other 9 contained DCIS only. A total of 32 of 36 (88.9%) of invasive carcinoma cases also had areas of DCIS. A total of 41 of 45 (91.1%) of all cases contained DCIS with 40 of 41 (97.6%) of those cases scored as high grade.

Morphological assessment was included for 60 young onset cases with a germline *BRCA1* pathogenic variant (group 3), 61 young onset cases with a germline *BRCA2* pathogenic variant (group 4) and 98 young onset breast cancer cases (group 5) with no identifiable germline high risk pathogenic variant (*BRCA1*, *BRCA2* or *TP53*) 21.

### Morphological review of breast tumours derived in germline TP53 carriers

The five young onset breast cancer cohorts were compared. Descriptive summary statistics are provided in Table 2.

**Table 2:** Morphological review comparing subgroups: morphological features in invasive breast tumour groups 1–5

Morphological feature		Subgroup				
Feature	Grading	*TP53*	HER2+	*BRCA1*	*BRCA2*	YBC
Tumour grade	1	2/36 (5.6%)	1/55 (1.8%)	7/138 (5.1%)	13/157 (8.3%)	35/217 (16.1%)
	2	16/35 (44.4%)	26/55 (47.3%)	33/138 (23.9%)	80/157 (51.0%)	86/217 (40.0%)
	3	18/36 (50.0%)	28/55 (50.9%)	98/138 (71.0%)	64/157 (40.8%)	96/217 (44.2%)
Tumour border	Pushing	0/36 (0.0%)	3/55 (5.4%)	80/138 (58.0%)	63/157 (40.1%)	94/217 (43.3%)
	Infiltrative	36/36 (100.0%)	52/55 (94.6%)	58/138 (42.0%)	93/157 (59.2%)	122/217 (56.2%)
	Missing	0/36 (0.0%)	0/55 (0.0%)	0/138 (0.0%)	1/157 (0.6%)	1/217 (0.5%)
Lymphocytic infiltration	Absent	14/36 (38.9%)	10/55 (18.2%)	13/138 (9.4%)	41/157 (26.1%)	58/217 (26.7%)
	Mild	16/36 (44.4%)	35/55 (63.6%)	59/138 (42.8%)	67/157 (42.7%)	96/217 (44.2%)
	Prominent	6/36 (16.7%)	10/55 (18.2%)	66/138 (47.8%)	48/157 (30.6%)	63/217 (29.0%)
	Missing	0/36 (0.0%)	0/55 (0.0%)	0/138 (0.0%)	1/157 (0.6%)	0/217 (0.0%)
Vascular invasion	Absent	24/36 (66.7%)	36/55 (65.4%)	118/138 (85.5%)	115/157 (73.2%)	168/217 (77.4%)
	Present	12/36 (33.3%)	19/55 (34.6%)	20/138 (14.5%)	39/157 (24.8%)	43/217 (19.8%)
	Missing	0/36 (0.0%)	0/55 (0.0%)	0/138 (0.0%)	3/157 (1.9%)	6/217 (2.8%)
Tumour stroma	Cellular	1/36 (2.8%)	6/55 (10.9%)	26/138 (18.8%)	23/157 (14.6%)	33/217 (15.2%)
	Sclerotic	29/36 (80.6%)	28/55 (50.9%)	50/138 (36.2%)	65/157 (41.4%)	95/217 (43.8%)
	Desmoplastic	6/36 (16.7%)	20/55 (36.4%)	39/138 (28.3%)	34/157 (21.7%)	63/217 (29.0%)
	Myxoid	0/36 (0.0%)	1/55 (1.8%)	3/138 (2.2%)	11/157 (7.0%)	6/217 (2.8%)
	Other	0/36 (0.0%)	0/55 (0.0%)	19/138 (13.8%)	23/157 (14.6%)	20/217 (9.2%)
	Missing	0/36 (0.0%)	0/55 (0.0%)	1/138 (0.7%)	1/157 (0.6%)	0/217 (0.0%)

Missing data refers to an unreported feature.

**Figure 1:**
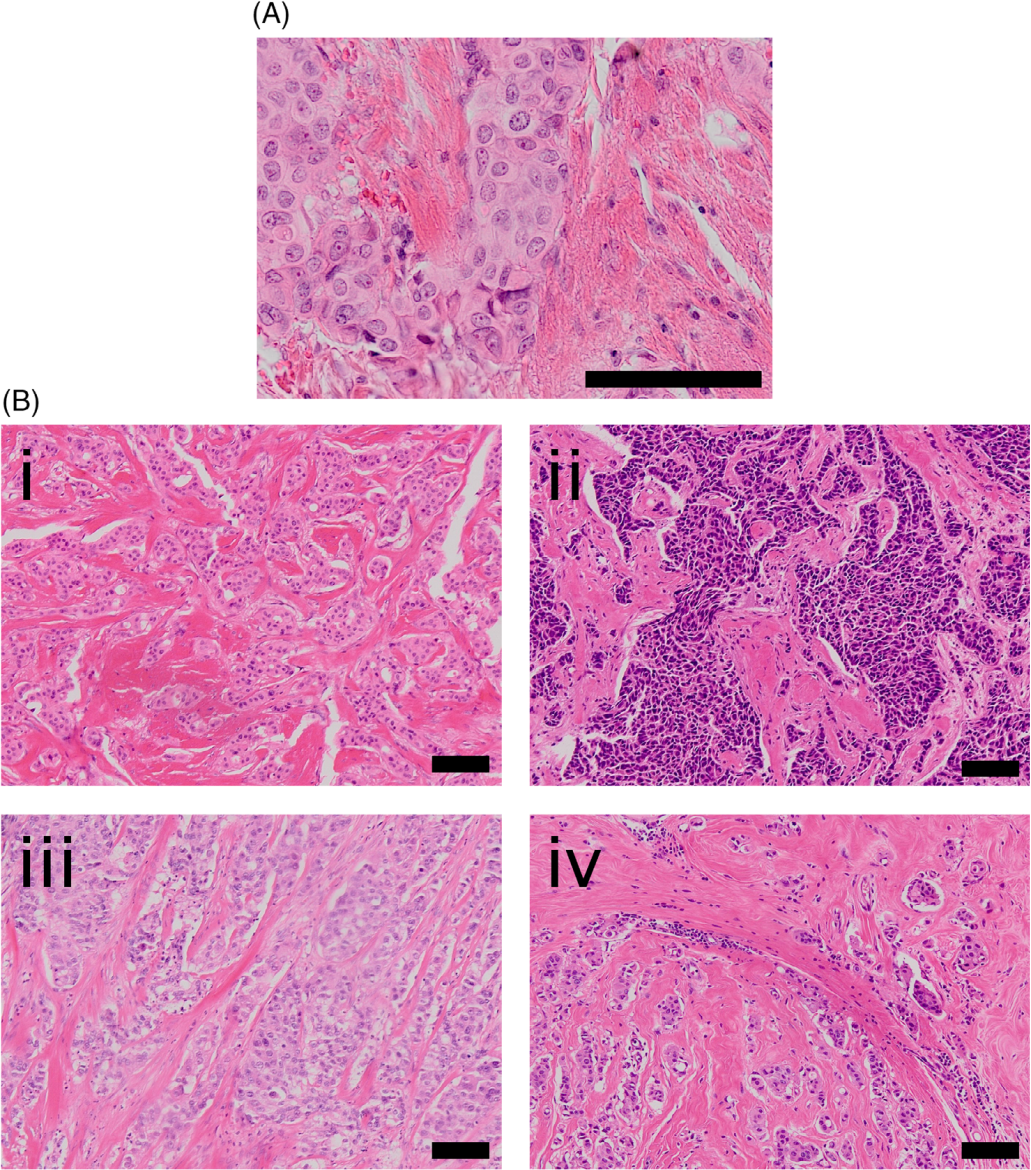
Sclerotic stroma in *TP53* carriers. (A) A magnified area of invasive tumour surrounded by sclerotic stroma. (B) (i)–(iv) Four patient samples with invasive carcinoma surrounded by a sclerotic stroma. Images were taken on the Olympus Dotslide at an objective magnification of ×20 (A) or ×10 (B). Scale bars represent 100 μm.

**Figure 2:**
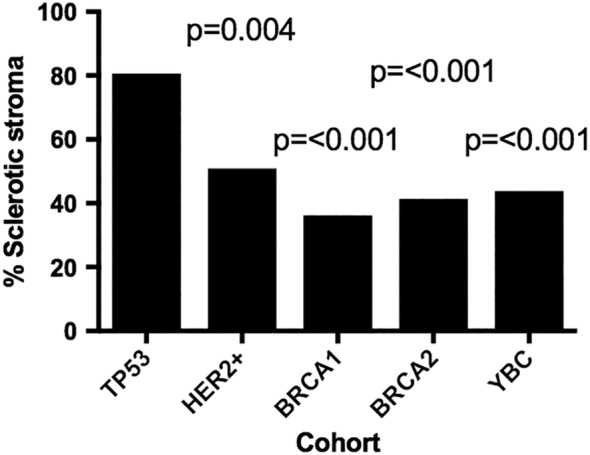
*TP53* carriers had a significantly higher proportion of sclerotic tumour stroma. The bar chart shows the frequencies of sclerotic stroma between cohorts. Statistics were performed on *TP53* carriers against each of the other groups using the Pearson Chi‐square test. Missing data were excluded.

A low level of lymphocytic infiltration was reported in both *TP53* carriers and HER2 positive cases (absent/mild lymphocytic infiltration): *TP53* 30 of 36 (83.3%), HER2 positive 45 of 55 (81.8%). This particularly contrasts with the *BRCA1* carriers where lymphocytic infiltration is a well‐recognised feature with a significantly lower proportion reported as having absent or mild lymphocytic infiltrate, 72 of 138 (52.2%). *BRCA2* carriers were similar to YBC, non‐carriers with 108 of 157 (68.8%) and 154 of 217 (71.0%) respectively. The tumour border was more often infiltrative in the *TP53* cohort, 36 of 36 (100.0%), and amongst HER2 positive cases, 52 of 55 (94.5%), compared to the *BRCA1*, *BRCA2* and YBC cases – 58 of 138 (42.0%), 93 of 157 (59.2%), 122 of 217 (56.2%) respectively. Vascular invasion was more frequently reported as ‘present’ in cases which were *TP53* 12 of 36 (33.3%) and HER2 positive 19 of 55 (34.5%) compared to *BRCA1*, *BRCA2* and YBC – 20 of 138 (14.5%), 39 of 157 (24.8%) and 43 of 217 (19.8%) respectively. The most striking morphological feature distinguishing the *TP53* germline carriers was the presence of a densely sclerotic tumour stroma (Figure 1) reported in 29 of 36 (80.6%) of cases, compared with 28 of 55 (50.9%) of young patients with HER2 positive breast cancer with no germline *TP53* pathogenic variant (*p* = 0.004) and lower still in other groups; 50 of 138 *BRCA1* carriers (36.2%, *p* < 0.001), 65 of 157 *BRCA2* carriers (41.4%, *p* < 0.001) and 95 of 217 in YBC (43.8%, *p* < 0.001) (Table 3 and Figure 2).

**Table 3:** The distribution of stromal types in YBC onset cohorts

Cohort	Sclerotic – (% of cohort)	Sclerotic + (% of cohort)	Missing data
*TP53*	7/36 (19.4%)	29/36 (80.6%)	0/36 (0.0%)
HER2+	27/55 (49.1%)	28/55 (50.9%)	0/55 (0.0%)
*BRCA1*	87/138 (63.0%)	50/138 (36.2%)	1/138 (0.7%)
*BRCA2*	91/157 (58.0%)	65/157 (41.4%)	1/157 (0.6%)
YBC	122/217 (56.2%)	95/217 (43.8%)	0/217 (0.0%)

### Receptor status in TP53 carriers

Hormone receptor status was evaluated and compared to available data in young onset breast cancer cohorts between *TP53* gene carriers (*n* = 36) and data available from the POSH cohort (*n* = 1260). *TP53* gene carriers were significantly more likely to be ER+/PR+/HER2+ (*p* < 0.001) than in the POSH cohort (Table 4).

**Table 4:** Receptor status compared with POSH cohort data

Tumour receptor status	COPE cohort, *TP53* gene carriers (%) *n* = 36	POSH cohort (%) *n* = 1260	*P* value*
HER2+/ER+/PR+	13/36 (36.1%)	145/1260 (11.5%)	<0.001
HER2+/ER+/PR−	1/36 (2.8%)	34/1260 (2.7%)	ns
HER2+/ER−/PR+	0/36 (0.0%)	10/1260 (0.8%)	ns
HER2+/ER−/PR−	6/36 (16.7%)	97/1260 (7.7%)	0.023
HER2−/ER+/PR+	7/36 (19.4%)	477/1260 (37.9%)	ns
HER2−/ER+/PR−	2/36 (5.6%)	77/1260 (6.1%)	ns
HER2−/ER−/PR+	0/36 (0.0%)	27/1260 (2.1%)	ns
HER2−/ER−/PR−	3/36 (8.3%)	393/1260 (31.2%)	0.008
Missing data	4/36 (11.1%)	0/1260 (0.0%)	

Summary statistics for receptor status in invasive breast tumours within *TP53* carriers and the YBC onset cohort POSH. Missing data refers to an unreported feature.

* Fisher's exact test.

### Stromal markers: αvβ6 and α‐SMA in *TP53* carriers

**Figure 3:**
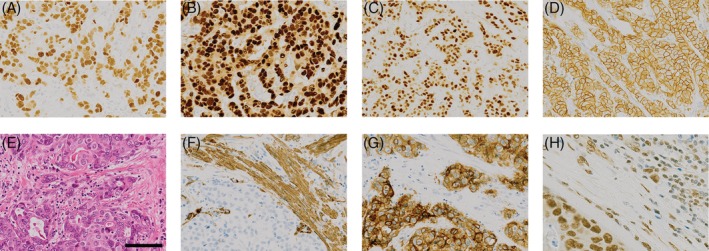
Examples of typical IHC for breast tumours arising in a mutant *TP53* background. Tumours are typically ER (A), PR (B) and HER2 (D) positive, show strong nuclear p53 staining (C) and are positive for markers of activated TGFβ signalling (F, αSMA; G, integrin αvβ6; H, pSMAD2/3). A corresponding H&E stain is shown in (E).

A high number of *TP53* carriers showed strong expression of p53 protein (≥5+: 69.4%, 25/36). Additionally, moderate to high expression of the stromal marker integrin αvβ6 was confirmed in 21 of 36 (58.3%) of the invasive tumours of *TP53* carriers in this study. High expression of α‐SMA (88.9%, 32/36) and pSMAD2/3 (proportion 3/4: 30/36; 83.3%, intensity 2/3: 29/36; 80.6%) were confirmed in a high proportion of tumours (Figure 3).

We explored whether the type of germline *TP53* pathogenic variant present amongst the COPE cases with invasive tumour altered the tumour phenotype. Comparing missense (*n* = 24), with truncating (*n* = 12) variants, excluding missing cases, a missense pathogenic variant did appear more likely to be associated with a sclerotic stroma (21/24, 87.5%) than a truncating pathogenic variant (8/12, 67.7%) although this was not statistically significant. HER2 overexpression was slightly less frequent with germline missense (12/24, 50.0%) versus truncating (8/12, 66.7%) variants but again the difference was not statistically significant.

Finally, we looked at the additional morphological data for POSH cases with all four IHC markers (Table 5). Amongst the 1260 young cancers from which TMA IHC scores were available, aberrant p53 staining was present in 302. Of these, 65 also had morphology evaluated in the previous study 22. Sclerotic stroma was reported in 26 of 65 (40.0%) of cases; 3 of 26 (11.5%) were also HER2‐positive but none were ER/PR/HER2 positive. Desmoplastic stroma was reported in 21 of 65 (32.3%) of cases; 5 of 21 (23.8%) of these were also HER2+. Cellular stroma was reported in 6 of 65 (9.2%) cases, all of which were triple negative.

**Table 5:** Expression of p53 and stromal markers in *TP53* gene carrier breast cancers

Stain	Scoring		*TP53* gene carriers
p53 (0–7)	0–1		0/36 (0.0%)
	2–4		7/36 (19.4%)
	5+		25/36 (69.4%)
	Missing data		4/36 (11.1%)
Integrin αvβ6	Absent/low		11/36 (30.6%)
	Moderate/high		21/36 (58.3%)
	Missing data		4/36 (11.1%)
α‐SMA	Absent/low		1/36 (2.8%)
	Moderate/high		32/36 (88.9%)
	Missing data		3/36 (8.3%)
pSMAD2/3	Staining proportion	1	1/36 (2.8%)
		2	0/36 (0.0%)
		3	7/36 (19.4%)
		4	23/36 (63.9%)
	Staining intensity	1	2/36 (5.6%)
		2	20/36 (55.6%)
		3	9/36 (25.0%)
	Missing data		5/36 (13.9%)

Amongst POSH cases 286 of 1260 (20.3%) were HER2 amplified and of these just over half (145) were ER/PR/HER2 positive (Table 4). We had information about stromal morphology in 121 of 1260 cases with complete IHC; 42 of 121 (34.7%) were HER2 positive, only 10 of these were also p53 positive. The stroma in these 10 cases was reported as desmoplastic (5), sclerotic (3), myxoid (2) and other (1). Only two cases were positive for ER/PR/HER2 and p53.

## Discussion

Patients with a germline *TP53* pathogenic variant typically develop high grade (2 or 3), HER2 positive, infiltrating ductal carcinoma, confirming our previously reported observation and subsequent reports from other groups 11, 12, 13. The frequency of triple positivity (HER2, ER and PR) also appears significantly higher in these patients than young onset cases in general. A number of other morphological features are similar between the cancers in germline *TP53* pathogenic variants and the HER2 positive early onset cases from the POSH study. However, the high frequency of breast tumours with densely sclerotic tumour stroma is a novel observation in patients with a germline *TP53* pathogenic variant. In comparison to sporadic HER2 amplified young onset breast cancers evaluated using the same methodology, the frequency of densely sclerotic stroma was striking. In comparison, the frequency of sclerotic stroma in sporadic HER2 positive cases was more similar to the proportion reported in other young onset cases; although we recognise that the data from the other young onset groups was taken from a previously reported study so may be less directly comparable. Amongst sporadic HER2 amplified tumours with abnormal p53 expression, there was no obvious excess of sclerotic stroma reported, although numbers were relatively small.

In sporadic breast cancers, somatic *TP53* mutations are most frequent in triple negative and HER2 positive tumours, less frequent in ER positive or HER2 negative cancers. Loss of function in the tumour tissue occurs through inactivation or loss of both alleles. Patients with breast cancer arising on a background of an inherited *TP53* pathogenic variant have already developed the first hit along the molecular pathway to carcinoma. It is possible that very early loss of *TP53* function through somatic mutation in patients with an inherited *TP53* pathogenic variant may be the reason for the more frequent development of the sclerotic stroma.

We explored this further by comparing the type of inherited variant. Pathogenic missense variants in *TP53* often disrupt the function of the wild type protein (dominant negative effect); this leads to loss of p53 function, even before the evolving cancer cell develops loss or amplification of genomic material, and often leads to a more severe Li–Fraumeni phenotype 23, 24, 25, 26, 27, 28. We hypothesise that a germline pathogenic missense variant would be more likely to be associated with a dense stromal reaction if this early loss of function was the underlying driving factor. Although numbers of cases were quite small, we did observe a higher proportion of cases with dense sclerotic stroma amongst patients with missense pathogenic variants, lending some support to this hypothesis.

There is increasing evidence to suggest that the intricate tumour‐stromal interactions in the TME are essential to driving tumour progression 16, 22, 23, 24, 25, 26. This complex system involves various cell types, including fibroblasts, immune cells and endothelial cells. The novel finding from this study was that breast tumours derived from germline *TP53* carriers typically presented with an associated sclerotic tumour stroma. Myofibroblasts are the cellular component of the microenvironment that deposit the rich collagen layer in a morphologically sclerotic stroma 27, 28, 29, 30. Myofibroblasts are characterised by α‐SMA expression 31. Tumours containing a high proportion of cancer‐associated fibroblasts (CAFs) positive for α‐SMA, have been associated with a poorer prognosis as a result of increased migration, invasion, proliferation, angiogenesis and inhibition of infiltrating lymphocytes 16, 22, 23, 24, 25. In *TP53* carriers, high expression of α‐SMA in the surrounding stroma was confirmed in 88.9% of cases, suggesting that CAFs are playing a key role.

A key pathway through which CAFs undergo transformation and activate tumour‐promoting processes is via transforming growth factor beta (TGFβ) signalling. One of the key mechanisms by which TGFβ is activated is through the expression of integrin αvβ6 on the cell surface of tumour cells 27. Integrin αvβ6 expression was confirmed in 58.3% of invasive *TP53* carrier tumours in this study, compared to only 15–16% of cases noted in a 2000 patient cohort reported elsewhere 32. PhosphoSMAD2/3 proteins are activated through a phosphorylation cascade, which forms the basis of initiated TGFβ signalling. Activated pSMAD2/3 proteins migrate from the cytoplasm to the nucleus and initiate transcription and the downstream deposition of collagen. Confirmation of TGFβ signalling was evidenced by high levels of pSMAD2/3 expression in breast cancers from *TP53* carriers.

Patients with inherited *TP53* pathogenic variants develop a broad range of tumours including sarcomas and childhood onset adrenocortical carcinomas; an estimated 50% of women develop breast cancer, usually at young ages. Treatment with chemotherapy and radiotherapy is thought to increase the risk of DNA damage‐induced late toxic effects, further increasing the risk of developing second malignancies. The invasive tumour characteristics in this group are typically associated with poor outcomes, including a low level of TILs in 83% of *TP53* carriers, with an infiltrative tumour border in 100% and vascular invasion in 33.3%. A potential mechanism by which TILs could be being blocked from migrating towards the tumour is the deposition of a collagen barrier by the CAFs 33, 34, 35, 36, 37, 38. Furthermore, CAFs positive for α‐SMA have previously been reported to increase migration, invasion, proliferation and angiogenesis 26, 39, 40.

Work by other groups has previously implicated p53 in the upregulation of collagen 41. Loss of p53 function was shown to upregulate TGFβ signalling and as a consequence lead to the transcriptional activation of COL1A2 and collagen synthesis 41. Murine models heterozygous for loss of *TP53* function (p53^+/−^) developed an extensive proliferative stromal reaction that was positive for α‐SMA and S100A4, a fibroblast marker 42. Finally, multiple groups have previously suggested that, through indirect cellular contact, tumour cells can inhibit wild‐type p53 activation and stimulate immunosuppression 43, 44.

In summary, we have observed that breast tumours arising in germline *TP53* pathogenic variant carriers are highly likely to be high grade, HER2 positive, ER/PR positive tumours with an associated dense sclerotic tumour stroma. The early inactivation of wild‐type p53 may be one of the important mechanisms leading to the activation of TGFβ via integrin αvβ6 and the development of breast cancers with many adverse prognostic characteristics. Given the increased risk of late toxicity from cytotoxic treatments (chemotherapy and radiotherapy) in germline *TP53* gene carriers, treatment with targeted agents including anti‐HER2 therapies such as trastuzumab (Herceptin) or antibodies targeting the TGFβ signalling pathway may be safer treatment options for breast cancer patients found to carry a germline *TP53* pathogenic variant.

## Author contributions statement

DME conceived and designed the study. KP, GM, MS, ES, KW, GT, AB and DME planned and executed the study. ES, LJ, LH, DGE, JB, AH and NP were responsible for sample acquisition. MS and GM conducted morphology reporting. GT, AB, GM and KM scored IHC data. KP, LH and LJ were responsible for TMA curation. All authors reviewed the manuscript, contributed to revisions and approved the final manuscript for submission.
